# Direct Measurement of the Reduced Scattering Coefficient by a Calibrated Random Laser Sensor

**DOI:** 10.3390/s22041401

**Published:** 2022-02-11

**Authors:** Federico Tommasi, Baptiste Auvity, Lorenzo Fini, Fabrizio Martelli, Stefano Cavalieri

**Affiliations:** 1 Dipartimento di Fisica e Astronomia, Università di Firenze, Via Sansone 1, 50019 Sesto Fiorentino, Italy; lorenzo.fini@unifi.it (L.F.); fabrizio.martelli@unifi.it (F.M.); 2 Département de Physique, Université Paris-Saclay, Bâtiment Hbar 625-Porte 333 Rue Louis de Broglie, 91405 Orsay, France; baptiste.auvity@gmail.com

**Keywords:** sensor, random laser, scattering

## Abstract

The research in optical sensors has been largely encouraged by the demand for low-cost and less or non-invasive new detection strategies. The invention of the random laser has opened a new frontier in optics, providing also the opportunity to explore new possibilities in the field of sensing, besides several different and peculiar phenomena. The main advantage in exploiting the physical principle of the random laser in optical sensors is due to the presence of the stimulated emission mechanism, which allows amplification and spectral modification of the signal. Here, we present a step forward in the exploitation of this optical phenomenon by a revisitation of a previous experimental setup, as well as the measurement method, in particular to mitigate the instability of the results due to shot-to-shot pump energy fluctuations. In particular, the main novelties of the setup are the use of optical fibers, a reference sensor, and a peristaltic pump. These improvements are devoted to: eliminating optical beam alignment issues; improving portability; mitigating the variation in pump energy and gain medium performances over time; realizing an easy and rapid change of the sensed medium. The results showed that such a setup can be considered a prototype for a portable device for directly measuring the scattering of liquid samples, without resorting to complicated numerical or analytic inversion procedures of the measured data, once the suitable calibration of the system is performed.

## 1. Introduction

Random lasing [1] is a physical phenomenon that generates a special kind of optical radiation, with mixed properties between common light and laser one. It is well known that a conventional laser is composed of three key elements: the pumping system, the gain medium, and the optical cavity. In a random laser system, the last of these three elements, which allows the radiation to spend a long enough time inside the gain medium for the amplification, is missing. To prevent the radiation from suddenly escaping the gain medium, a suitable level of disorder is introduced to such a material. Thus, if the induced scattering is strong enough, the optical radiation undergoes amplification by stimulated emission before exiting from the medium. Hence, the output radiation has the poor directionality typical of spontaneous emission, but the spectral narrowing of the stimulated one. Random lasing is a process whose temporal dynamics begins, in general, with a pump pulse that excites the gain medium; once the first emitted photons are generated by spontaneous emission, the energy stored in the medium, in the presence of enough scattering, undergoes depletion by the mechanism of stimulated emission. Hence, as in a conventional laser, for a random laser system, the threshold energy can be defined, in a nutshell, in the conditions that allow the gain to overcome the losses; the critical difference consists of the fact that the threshold in a random laser depends on the scattering strength, because, in the absence of an optical cavity, it is the mechanism that can support the feedback.

The main features of the random laser have been reported for the first time in the pioneering theoretical work of Letokhov [2] at the end of 1960s and experimentally realized since the 1990s in laser dye with nanoparticles [3], polymer films [4], organic media [5], laser crystal powder [6], cold atoms [7], semiconductor powder [8], dye-infiltrated biological tissue [9], optical fibers [10,11,12], stimulated Raman scattering [13], liquid crystals [14,15,16], plasmonics [17,18], dye-infiltrated opals [19], and perovskite [20]. The characteristics of the random laser have been extensively studied during the last three decades, and applications have been proposed, in particular in the fields of sensing [21,22,23,24,25,26], illumination [27,28,29,30], spectroscopy [31], optical networks [32,33], the statistics of events and fluctuations [34,35,36,37,38,39,40,41,42,43], replica symmetry breaking phenomenology [44,45,46,47,48,49,50,51], anomalous diffusion [52,53], turbulence [54], and chemical properties’ characterization [55,56].

Since the random laser emission strongly depends on the scattering, such an optical system has been considered a promising strategy for sensing the scattering strength of a material. Unlike a conventional laser, in this case, the scattering is not a loss factor, and it is instead fundamental to provide the feedback mechanism and thus to “feed” the signal. Because of the spectral narrowing of the spectrum due to stimulated emission, by adding gain properties to a scattering medium, the analysis of the emission can lead to a measure of the concentration of the scatterers. Hence, random-laser-based sensors can be framed in the field of *active sensors*, where the term “active” can be used to describe a sensor whose working principle is based on stimulated emission, whereas in the “passive” conventional case, such a mechanism is absent. Such passive methods cover several fields of research in the diagnostics of biological tissue [57,58]. The stimulated emission provides both the signal amplification and the spectral modifications that can carry information about the sample properties. The possibility to follow a sensing strategy based on a random laser is also considered of particular interest in biomedical optics because biological tissues can be described as a scattering medium for visible and NIR radiation. Indeed, random lasing has been reported in biological tissues infiltrated by dye molecules, such as animal tissue [59], bone [60], and the wings of insects [61,62]. Moreover, ex vivo cancerous tissue, once infiltrated by a gain material, has been reported as suitable to generate random lasing, opening new opportunities in diagnostics [63,64,65] and opto-chemical therapies [66]. In fact, malignant tissue shows a different spectral signature in the random laser emission compared to a healthy one, due to differences in the microstructure.

In using random laser systems to investigate diffusive samples, the main drawback in practical applications is the alteration of the sample by the gain material. Another issue is the requirement to direct pumping at the sample, causing possible optically induced thermal damages. Even though bio-compatible materials have been reported [67,68,69,70], in possible in vivo applications in medicine, the toxicity of the gain material is a critical issue, since, for instance, the typical laser dye molecules have cancerous properties.

A solution for a non-invasive sensor has been found in the separation between the gain material and the scattering one [22], with a device composed of a transparent glass hollow sphere combined with an optical fiber. A gain material is inserted in the sphere, in general an alcoholic solution of a dye, with the addition of a small quantity of nanoparticles as scatterers. The hollow sphere of glass, around 2 mm diameter, has transparent walls that are intrinsically partially reflective because of the refractive index mismatch between the glass and the internal/external environment. The proper amount of nanoparticles introduces the suitable scattering strength to prevent both laser action due to the sensor itself and the intrinsic random laser. The optical fiber carries both the pump pulse, provided by a pulsed laser, and the random laser signal. Such a detector shows a high sensitivity for the variation of the scattering strength [22], as well as the possibility to reveal the scatterers’ size [23].

**Figure 1:**
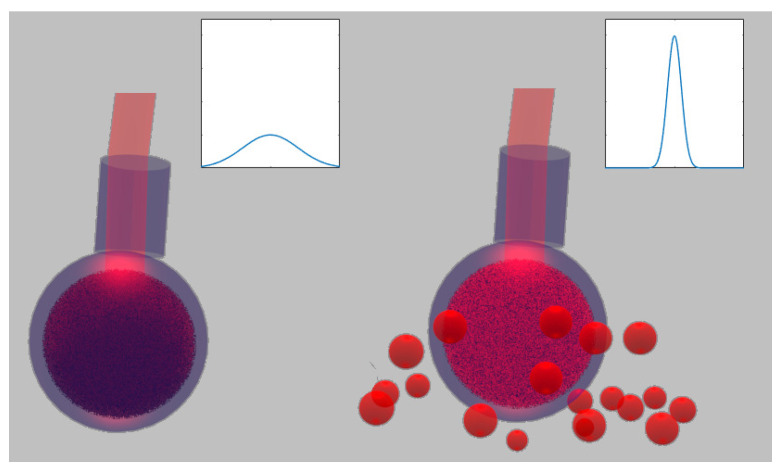
Working principle of the random laser sensor with external feedback. Given the same pumping energy, only once the sensor is put into contact with an external scattering sample, the random laser emission occurs. The insets represent a picture of the shape of the possible emission spectra. On the left, without the external feedback provided by external scatterers, the spectrum of the signal is broad and of low intensity, because the energy simply escapes from the sensor. On the right, the feedback allows a larger amount of light to be collected back by the fiber, whereas the spectrum becomes narrower due to the stimulated emission.

The simple pictures shown in Figure 1 show the working principle of the random laser sensor based on external feedback. Without an external material close to the sensor, the emission is the one typical of spontaneous emission, since the disorder inside the sensor is not strong enough to trigger random lasing for any pump energy injected. Only once the sensor is put into contact with an external medium, the material inside the sensor can show an over-threshold behavior. Indeed, the light, initially emitted by spontaneous emission inside the gain material, can propagate outside the glass sphere and, after some scattering events, can reach again the gain material, inducing stimulated emission. Hence, the scattering external sample provides a critical feedback to reach the random laser threshold. The geometry of this sensor is suitable in particular for analyzing liquid diffusive samples, where it can be immersed to collect a large amount of feedback light, but it can also work once put into contact with a solid sample, such as powders or scattering surfaces.

Here, we present a further optimization of such a non-invasive detection system by the implementation of a system of fibers, which allows the simultaneous analysis of two sensors, and a mechanism for injection and removal of liquid samples as well. This system can guarantee a higher stability and reproducibility of the measurement, and it also represents a prototype for a fast, stable, and portable device that does not need critical alignment of the optical beams.

The results here shown involve the direct measurement of the reduced scattering coefficient $\mu_{s}^{\prime }$, which is a fundamental parameter to characterize the diffusive properties of a turbid medium. This quantity is linked to the scattering coefficient $\mu_{s}$, i.e., the reciprocal of the scattering mean free path, by the relation $\mu_{s}^{\prime }=\mu_{s}\left(1-g\right)$, where *g* is the asymmetry factor of the scattering function. Explicit calculation methods for $\mu_{s}^{\prime }$ were, for instance, reported in [23,71].

## 2. Experimental Setup

The sensors reported in [22,23] have led to new challenges to improve the stability due to the use of dyes as the gain material. The use of this kind of material as the active medium, as a proof of principle, is not a mandatory choice, but it shows many advantages, such as a moderate pumping energy, and it is easy to use. The effects of the alteration of the sample, as well as the toxicity for biological samples are avoided by the separation provided by the hollow sphere. The drawbacks are found, in particular, for long time measurement sessions, when the photodegradation of the dye [72,73] can affect the reproducibility.

**Figure 2:**
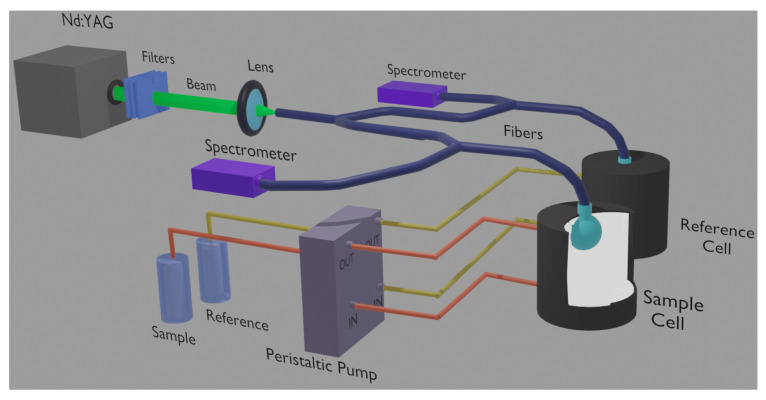
Scheme of the experimental setup. The sample cell is sectioned to allow seeing the sensor and the liquid sample inside.

The new experimental setup here presented, shown in Figure 2, has as a first goal to overcome such defects comparing the signals from twin sensors, one in contact with the sample and the other with a reference. In addition to the same aging and degradation that affect both sensors, the comparison between the two signals generated from the same pumping laser pulse can strongly mitigate the effects of the laser shot-to-shot intensity fluctuations in the precision and accuracy of the measurement.

Moreover, another problem for the reproducibility of the measurement is the placement of the sensor with respect to the sample, with a consequent variation of the signal. In this new setup, we overcame this issue with the special design described in the following.

In order to make such a working principle possible, the whole experimental setup of [22,23] was deeply changed, also achieving the portability provided by a system entirely composed of optical fibers. Both sensors were composed of a hollow glass sphere with a diameter of 3.5±0.2 mm and a stem 20 mm long with a diameter of 1.5 mm. The twin sensors were inserted into hermetically sealed black cylindrical cells of polyvinyl chloride, which allowed an easy change of the sample, by a peristaltic pump and ensured that the sensor was in any case immersed in the sample without further adjustments. Each cell had a height of 2 cm and a diameter of 1 cm, and two holes were present for the insertion of the metallic stems, which allowed the connection with the peristaltic pump by plastic tubes. A cap of 5 mm in height also had a central hole that helped to assemble the sensor. Different kinds of dye (rhodamine 640, rhodamine 590, rhodamine 610) and solvents (ethanol, ethylene glycol, methanol) were used inside the sensor for the different tests. The nanoparticles put inside the active medium were composed of ZnO or latex, and their concentration was high enough to introduce a small loss factor to prevent spontaneous laser-like oscillation with the refractive index mismatch between the solvent and the glass and also low enough to avoid random laser emission without the external feedback provided by the scattering sample. The concentration of the dye was empirically tuned to have different ranges of sensitivity for the $\mu_{s}^{\prime }$ of the external sample.

The scheme of the experimental setup, designed to analyze liquid samples of scattering materials, is shown in Figure 2. The pumping laser beam, provided by a frequency-doubled Q-switched Nd:YAG laser, after attenuation by filters, was focused into the entrance of an optical fiber that was split into two different paths and then toward two different sensors. Such a focusing was the only optical alignment requested in this new setup. Each laser pulse with an energy of around 1 mJ was sent to the sensors every 2 s. The liquid inside the reference cell remained the same for the whole set of measurements, whereas the one in the sample cell could be easily injected and removed by a peristaltic pump. Eventually, the signals produced by the sensors were injected back to the fibers to reach two different spectrometers, which were temporally activated and synchronized by the same trigger signal produced by the Nd:YAG laser. The signals acquired by the spectrometers (an Ocean Optics USB200+ for the reference and an Ocean Insight Flame for the sample) were compared shot-to-shot. Such a kind of analysis allowed improving the sensitivity and led to a comparable temporal degradation of the dye inside the sensor, as well as the shot-to-shot fluctuation of the pumping energy and the temperature of the environment.

The first phase consisted of the optimization of the setup by using the same sample in both cells, to adjust small variations in the pumping energy and in the signal sensitivity of the spectrometers by SMA optical attenuators (not shown in the figure). The next one involved calibration by means of a well-characterized medium to achieve the system response for different values of $\mu_{s}^{\prime }$. The peak of the random laser signal detected by this kind of sensor showed a dependence on $\mu_{s}^{\prime }$ also when the material of the scatterers was changed [23]. The optimal reference samples for the calibration here used were different water dilutions of Intralipid, which is a fat emulsion with an extensive characterization in the literature [74,75,76,77,78].

The $\mu_{s}^{\prime }$ values of the samples of the Intralipid were calculated for the wavelength of the peak of the emission spectrum of the used dye inside the sensor [76,78]. Once having obtained the calibration of the system, we tested the possibility of a direct measure of the $\mu_{s}^{\prime }$ of a liquid sample, as shown in the following.

## 3. Results

**Figure 3:**
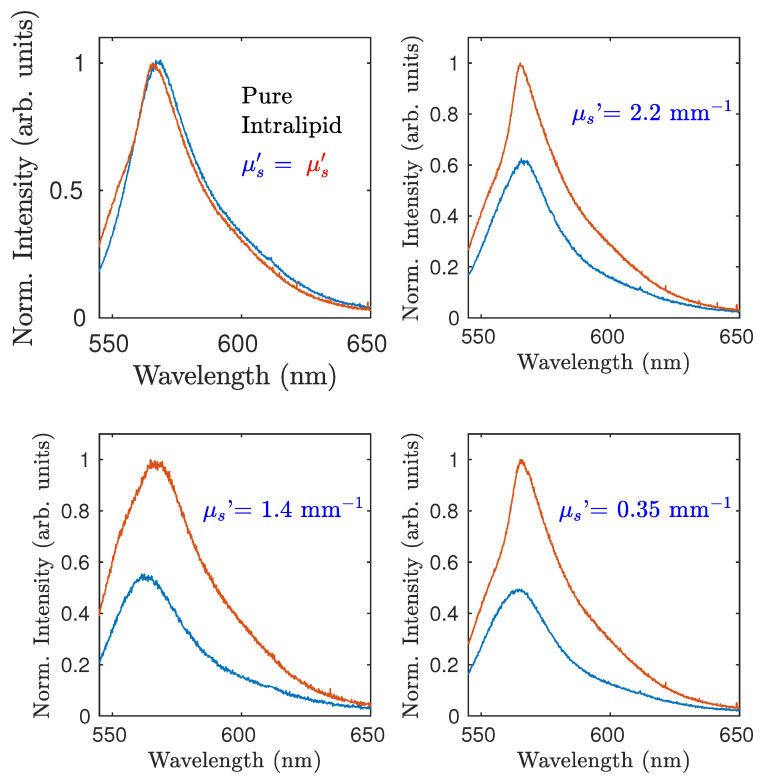
The red emission is for the sensor that always remains in contact with pure Intralipid, whereas the blue one pertains to different dilutions of Intralipid 20% (zero dilution for the top-left case).

In Figure 3, four spectra are shown for a couple of twin sensors. The top-left spectrum pertains to the case where both the black cells were filled with pure Intralipid 20%. The attenuators of the fibers were adjusted to have the same peak value of both emission spectra. The different shapes of the spectra were mainly due to small residual differences in the fabrication of the sensors.The other three shown spectra pertain to different examples of dilutions of the Intralipid (blue line) for a sensor, whereas the other one (red line) was always put in contact with pure Intralipid. Each spectrum of the sample was normalized to the correspondent spectrum provided by the reference given by the same pump pulse. Each value reported was averaged upon 10 shots. Such a procedure allowed taking into account the shot-to-shot variation of the pump beam energy, as well as long-term variations due to the dye degradation and the room temperature changes. Usually, the dye inside a sensor does not need to be changed for several days.

**Figure 4:**
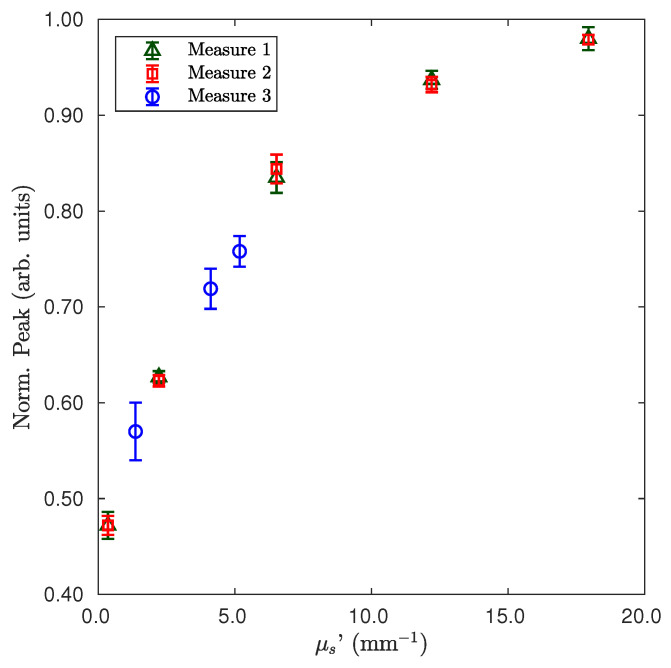
Test of reproducibility by measurements performed at different times and with two different sets of dilutions of Intralipid (one set for Measures 1 and 2 and the second one for Measure 3).

A reproducibility test of the measurement was performed over time and different realizations of the sample. In Figure 4, we report values for three measurements performed at different times (several hours of time lag) and with two different sets of Intralipid dilutions. The results are reported in Figure 4, where the same couple of sensors of Figure 3 were used with the same normalization procedure. The values reported refer to three different sets of measurements performed at different times and on two different sets of Intralipid dilutions. Each value is given by 10 shots, and the displayed uncertainty is the standard error. Such measurements were performed with the aim to show the stability of the system over time and then the robustness of the method in mitigating the effects of pump fluctuations, dye degradation, and sample changes. Moreover, for such a couple of sensors, an approximately linear zone was detectable up to $\mu_{s}^{\prime }≃$ 8 mm${}^{-1}$, whereas over such a value of the reduced scattering coefficient, the sensitivity appeared to decrease. Changing the property of the gain medium inside the sensor, the range of higher sensitivity of $\mu_{s}^{\prime }$ can be shifted.

**Figure 5:**
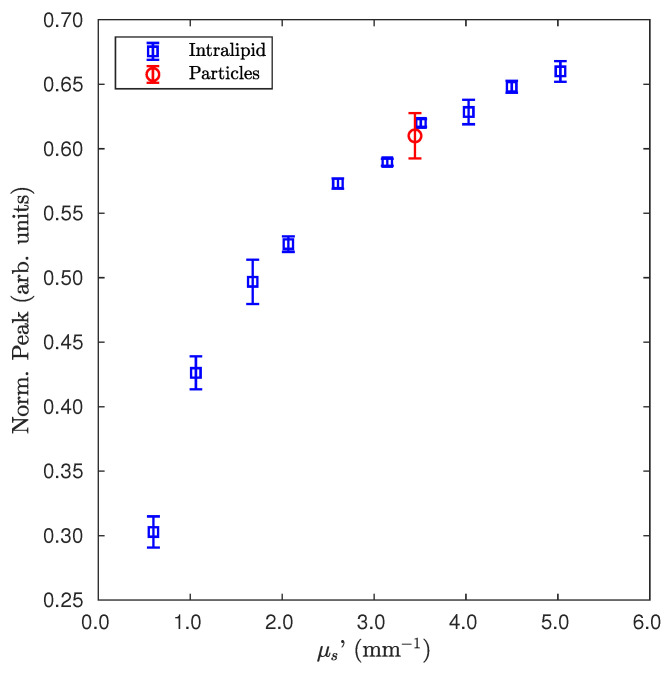
Measure of Intralipid of different dilutions (blue squares) and for particles of diameter 159 nm (red circle).

The results reported in Figure 5 pertain to a different couple of sensors and refer to a method devoted to measuring the $\mu_{s}^{\prime }$ of an unknown scattering sample by using a calibrated couple of sensors. Such a measurement provides a proof of principle of the direct detection of $\mu_{s}^{\prime }$ of an unknown sample. As a test, a sample to measure a water dispersion of latex nanoparticles was prepared. Such particles had a mean diameter of 159 nm and a concentration that theoretically led to a $\mu_{s}^{\prime }=3.4$ mm${}^{-1}$, numerically calculated by the Mie theory [23,71]. The test was performed by the measure of different dilutions of Intralipid (blue squares). The couple of sensors had a suitable dye concentration able to give a higher sensitivity in the range around the expected value of $\mu_{s}^{\prime }$ of the latex nanoparticle sample (red circle). As the figure shows, the measure of $\mu_{s}^{\prime }$ of the particles was consistent with the ones of the Intralipid dilutions.

## 4. Discussion

In this paper, a new experimental setup and method are reported for random laser “active” sensors based on external feedback and described for the first time in [22]. Such an experimental realization, suitable for liquid scattering samples, was based on a couple of identical sensors put inside a cell, where the samples were injected and removed by a peristaltic pump. The use of one of the sensors as the reference allowed mitigating the effect of the pump pulse fluctuations and the dye degradation over time.

Compared to similar measurements reported in [23], the setup showed substantial improvements, in particular concerning the speed of the measure, the simplicity of the experimental setup, and the long-term stability. In particular, it is worth stressing the achieved higher reproducibility over time and over different realizations of the samples, as well as the higher velocity in performing the measurement, thanks to the peristaltic pump for changing the sample, leaving fixed the sensor position. Hence, we think that the results herein reported showed the evident robustness of this new experimental setup. The requirement to build pairs of sensors with quite similar characteristics is not a critical point even for home-made devices, as the results herein reported showed. Furthermore, a substantial improvement in this aspect can be obtained by implementing industrial-like manufacturing.

The test shown in Figure 5 suggests that a calibrated system can provide a direct measure of the reduced scattering coefficient $\mu_{s}^{\prime }$ of an unknown scattering sample, since the height of the peak value can be directly associated with such a value, without successive data elaboration. Compared to systems that measure $\mu_{s}^{\prime }$ with “passive sensors”, in addition to the advantages mentioned above, the possibility to perform the measure over a very small quantity of material represents another strong point.

We believe that such an experimental setup can be considered a prototype of new portable systems based on “active sensors”, with very interesting practical applications in different fields, such as diagnostics, quality control, and material characterization.

## Data Availability

The datasets used and/or analysed during the current study available from the corresponding author on reasonable request.

## References

[1] Wiersma D.S. (2008). The physics and applications of random lasers. Nat. Phys..

[2] Letokhov V.S. (1968). Generation of Light by a Scattering Medium with Negative Resonance Absorption. Sov. J. Exp. Theor. Phys..

[3] Lawandy N.M., Balachandran R.M., Gomes A.S.L., Sauvain E. (1994). Laser action in strongly scattering median. Nature.

[4] Polson R.C., Vardeny Z.V. (2005). Organic random lasers in the weak-scattering regime. Phys. Rev. B.

[5] Frolov S.V., Vardeny Z.V., Yoshino K., Zakhidov A., Baughman R.H. (1999). Stimulated emission in high-gain organic media. Phys. Rev. B.

[6] Noginov M.A., Noginova N.E., Caulfield H.J., Venkateswarlu P., Thompson T., Mahdi M., Ostroumov V. (1996). Short-pulsed stimulated emission in the powders of NdAl3(BO3)4, NdSc3(BO3)4, and Nd:Sr5(PO4)3F laser crystals. J. Opt. Soc. Am. B.

[7] Froufe-Pérez L.S., Guerin W., Carminati R., Kaiser R. (2009). Threshold of a Random Laser with Cold Atoms. Phys. Rev. Lett..

[8] Cao H., Zhao Y.G., Ho S.T., Seelig E.W., Wang Q.H., Chang R.P.H. (1999). Random Laser Action in Semiconductor Powder. Phys. Rev. Lett..

[9] Wang L., Liu D., He N., Jacques S.L., Thomsen S.L. (1996). Biological laser action. Appl. Opt..

[10] Turitsyn S.K., Babin S.A., El-Taher A.E., Harper P., Churkin D.V., Kablukov S.I., Ania-Castañón J.D., Karalekas V., Podivilov E.V. (2010). Random distributed feedback fiber laser. Nat. Photonics.

[11] Zhai T., Niu L., Cao F., Tong F., Li S., Wang M., Zhang X. (2017). A RGB random laser on an optical fiber facet. RSC Adv..

[12] Churkin D.V., Sugavanam S., Vatnik I.D., Wang Z., Podivilov E.V., Babin S.A., Rao Y., Turitsyn S.K. (2015). Recent advances in fundamentals and applications of random fiber lasers. Adv. Opt. Photon..

[13] Hokr B.H., Bixler J.N., Cone M.T., Mason J.D., Beier H.T., Noojin G.D., Petrov G.I., Golovan L.A., Thomas R.J., Rockwell B.A. (2014). Bright emission from a random Raman laser. Nat. Commun..

[14] Wiersma D.S., Cavalieri S. (2001). A temperature-tunable random laser. Nature.

[15] Tiwari A.K., Pattelli L., Torre R., Wiersma D.S. (2018). Remote control of liquid crystal elastomer random laser using external stimuli. Appl. Phys. Lett..

[16] Sznitko L., Kaliciak K., Adamow A., Mysliwiec J. (2016). A random laser made of nematic liquid crystal doped with a laser dye. Opt. Mater..

[17] Dice G.D., Mujumdar S., Elezzabi A.Y. (2005). Plasmonically enhanced diffusive and subdiffusive metal nanoparticle-dye random laser. Appl. Phys. Lett..

[18] Qiao Q., Shan C.X., Zheng J., Zhu H., Yu S.F., Li B.H., Jia Y., Shen D.Z. (2013). Surface plasmon enhanced electrically pumped random lasers. Nanoscale.

[19] Frolov S.V., Vardeny Z.V., Zakhidov A.A., Baughman R.H. (1999). Laser-like emission in opal photonic crystals. Opt. Commun..

[20] Shi Z., Sun X., Wu D., Xu T., Tian Y.T., Zhang Y., Li X.J., Du G. (2016). Near-infrared random lasing realized in perovskite CH3NH3PbI3 thin film. J. Mater. Chem. C.

[21] Choi S.H., Kim Y.L. (2014). The potential of naturally occurring lasing for biological and chemical sensors. Biomed. Eng. Lett..

[22] Ignesti E., Tommasi F., Fini L., Martelli F., Azzali N., Cavalieri S. (2016). A new class of optical sensors: A random laser based device. Sci. Rep..

[23] Tommasi F., Ignesti E., Fini L., Martelli F., Cavalieri S. (2018). Random laser based method for direct measurement of scattering properties. Opt. Express.

[24] Abegão L.M.G., Pagani A.A.C., Zílio S.C., Alencar M.A.R.C., Rodrigues J.J. (2016). Measuring milk fat content by random laser emission. Sci. Rep..

[25] Shi X., Ge K., Tong J.H., Zhai T. (2020). Low-cost biosensors based on a plasmonic random laser on fiber facet. Opt. Express.

[26] Hohmann M., Späth M., Ni D., Dörner D., Lengenfelder B., Klämpfl F., Schmidt M. (2021). Random laser as a potential tool for the determination of the scattering coefficient. Biomed. Opt. Express.

[27] Redding B., Choma M.A., Cao H. (2012). Speckle-free laser imaging using random laser illumination. Nat. Photonics.

[28] Chang S.W., Liao W.C., Liao Y.M., Lin H.I., Lin H.Y., Lin W.J., Lin S.Y., Perumal P., Haider G., Tai C.T. (2018). A White Random Laser. Sci. Rep..

[29] Shojaie E., Madanipour K., Bodermann B., Frenner K., Silver R.M. (2017). Detection of nanoparticle changes in nanocomposite active sample using random laser emission. Modeling Aspects in Optical Metrology VI.

[30] Xu Y., Zhang M., Lu P., Mihailov S., Bao X. (2016). Multi-parameter sensor based on random fiber lasers. AIP Adv..

[31] Boschetti A., Taschin A., Bartolini P., Tiwari A.K., Pattelli L., Torre R., Wiersma D.S. (2020). Spectral super-resolution spectroscopy using a random laser. Nat. Photonics.

[32] Giacomelli G., Lepri S., Trono C. (2019). Optical networks as complex lasers. Phys. Rev. A.

[33] Caselli N., Consoli A., Sánchez Á.M.M., López C. (2021). Networks of mutually coupled random lasers. Optica.

[34] Sharma D., Ramachandran H., Kumar N. (2006). Lévy statistical fluctuations from a random amplyfyng medium. Fluct. Noise Lett..

[35] Mujumdar S., Ricci M., Torre R., Wiersma D.S. (2004). Amplified Extended Modes in Random Lasers. Phys. Rev. Lett..

[36] Lepri S. (2013). Fluctuations in a Diffusive Medium with Gain. Phys. Rev. Lett..

[37] Lepri S., Cavalieri S., Oppo G.L., Wiersma D.S. (2007). Statistical regimes of random laser fluctuations. Phys. Rev. A.

[38] Uppu R., Tiwari A.K., Mujumdar S. (2012). Identification of statistical regimes and crossovers in coherent random laser emission. Opt. Lett..

[39] Ignesti E., Tommasi F., Fini L., Lepri S., Radhalakshmi V., Wiersma D.S., Cavalieri S. (2013). Experimental and theoretical investigation of statistical regimes in random laser emission. Phys. Rev. A.

[40] Tommasi F., Ignesti E., Fini L., Cavalieri S. (2015). Controlling directionality and the statistical regime of the random laser emission. Phys. Rev. A.

[41] Tommasi F., Fini L., Ignesti E., Lepri S., Martelli F., Cavalieri S. (2018). Statistical outliers in random laser emission. Phys. Rev. A.

[42] Uppu R., Mujumdar S. (2013). Dependence of the Gaussian-Lévy transition on the disorder strength in random lasers. Phys. Rev. A.

[43] Lima B.C., Pincheira P.I.R., Raposo E.P., Menezes L.d.S., de Araújo C.B., Gomes A.S.L., Kashyap R. (2017). Extreme-value statistics of intensities in a cw-pumped random fiber laser. Phys. Rev. A.

[44] Gofraniha N., Viola I., Di Maria F., Barbarella G., Gigli G., Leuzzi L., Conti C. (2015). Experimental evidence of replica symmetry breaking in random lasers. Nat. Commun..

[45] Antenucci F., Crisanti A., Leuzzi L. (2015). The glassy random laser: Replica symmetry breaking in the intensity fluctuations of emission spectra. Sci. Rep..

[46] Gomes A.S.L., Raposo E.P., Moura A.L., Fewo S.I., Pincheira P.I.R., Jerez V., Maia L.J.Q.d.A. (2016). Observation of Lévy distribution and replica symmetry breaking in random lasers from a single set of measurements. Sci. Rep..

[47] Tommasi F., Ignesti E., Lepri S., Cavalieri S. (2016). Robustness of replica symmetry breaking phenomenology in random laser. Sci. Rep..

[48] Araújo C.B.d., Gomes A.S.L., Raposo E.P. (2017). Lévy Statistics and the Glassy Behavior of Light in Random Fiber Lasers. Appl. Sci..

[49] Xia J., He J., Xie K., Zhang X., Hu L., Li Y., Chen X., Ma J., Wen J., Chen J. (2019). Replica Symmetry Breaking in FRET-Assisted Random Laser Based on Electrospun Polymer Fiber. Ann. Phys..

[50] Kong J., He J., Zhang J., Ma J., Xie K., Chen J., Hong L., Hu Z. (2021). Replica Symmetry Breaking in Cholesteric Liquid Crystal Bandgap Lasing. Ann. Phys..

[51] Gradenigo G., Antenucci F., Leuzzi L. (2020). Glassiness and lack of equipartition in random lasers: The common roots of ergodicity breaking in disordered and nonlinear systems. Phys. Rev. Res..

[52] Chen Y., Fiorentino A., Dal Negro L. (2019). A fractional diffusion random laser. Sci. Rep..

[53] Tommasi F., Fini L., Martelli F., Cavalieri S. (2019). Superdiffusive random laser. Phys. Rev. A.

[54] Roa González I., Lima B., Pincheira P., Brum A., Macêdo A., Vasconcelos G., Menezes L., Raposo E., Gomes A., Kashyap R. (2017). Turbulence Hierarchy in a Random Fibre Laser. Nat. Commun..

[55] Hokr B.H., Bixler J.N., Noojin G.D., Thomas R.J., Rockwell B.A., Yakovlev V.V., Scully M.O. (2014). Single-shot stand-off chemical identification of powders using random Raman lasing. Proc. Natl. Acad. Sci. USA.

[56] Gaio M., Caixeiro S., Marelli B., Omenetto F.G., Sapienza R. (2017). Gain-Based Mechanism for pH Sensing Based on Random Lasing. Phys. Rev. Appl..

[57] Martelli F., Del-Bianco S., Zaccanti G., Pifferi A., Torricelli A., Bassi A., Taroni P., Cubeddu R. (2004). Phantom validation and in vivo application of an inversion procedure for retrieving the optical properties of diffusive layered media from time-resolved reflectance measurements. Opt. Lett..

[58] Farina A., Torricelli A., Bargigia I., Spinelli L., Cubeddu R., Foschum F., Jäger M., Simon E., Fugger O., Kienle A. (2015). In-vivo multilaboratory investigation of the optical properties of the human head. Biomed. Opt. Express.

[59] Siddique M., Yang L., Wang Q., Alfano R. (1995). Mirrorless laser action from optically pumped dye-treated animal tissues. Opt. Commun..

[60] Song Q., Xiao S., Xu Z., Liu J., Sun X., Drachev V., Shalaev V.M., Akkus O., Kim Y.L. (2010). Random lasing in bone tissue. Opt. Lett..

[61] Zhang D., Kostovski G., Karnutsch C., Mitchell A. (2012). Random lasing from dye doped polymer within biological source scatters: The pomponia imperatorial cicada wing random nanostructures. Org. Electron..

[62] Wang C.S., Chang T.Y., Lin T.Y., Chen Y.F. (2014). Biologically inspired flexible quasi-single-mode random laser: An integration of Pieris canidia butterfly wing and semiconductors. Sci. Rep..

[63] Polson R.C., Vardeny Z.V. (2004). Random lasing in human tissues. Appl. Phys. Lett..

[64] Polson R.C., Vardeny Z.V. (2010). Cancerous tissue mapping from random lasing emission spectra. J. Opt..

[65] He J., Hu S., Ren J., Cheng X., Hu Z., Wang N., Zhang H., Lam R.H.W., Tam H.Y. (2019). Biofluidic Random Laser Cytometer for Biophysical Phenotyping of Cell Suspensions. ACS Sens..

[66] Lahoz F., Martín I.R., Urgellés M., Marrero-Alonso J., Marín R., Saavedra C.J., Boto A., Díaz M. (2015). Random laser in biological tissues impregnated with a fluorescent anticancer drug. Laser Phys. Lett..

[67] Umar M., Min K., Kim S., Kim S. (2019). Random lasing and amplified spontaneous emission from silk inverse opals: Optical gain enhancement via protein scatterers. Sci. Rep..

[68] Lin W.J., Liao Y.M., Lin H.Y., Haider G., Lin S.Y., Liao W.C., Wei R.T., Perumal P., Chang T.Y., Tseng C.Y. (2018). All-marine based random lasers. Org. Electron..

[69] Biswas S., Kumbhakar P. (2017). Continuous wave random lasing in naturally occurring biocompatible pigments and reduction of lasing threshold using triangular silver nanostructures as scattering media. Nanoscale.

[70] Gummaluri V.S., Krishnan S.R., Vijayan C. (2018). Stokes mode Raman random lasing in a fully biocompatible medium. Opt. Lett..

[71] Martelli F., Del Bianco S., Ismaelli A., Zaccanti G. (2009). Light Propagation through Biological Tissue and Other Diffusive Media: Theory, Solutions, and Software.

[72] Brito-Silva A.M., Galembeck A., Gomes A.S.L., Jesus-Silva A.J., de Araújo C.B. (2010). Random laser action in dye solutions containing Stöber silica nanoparticles. J. Appl. Phys..

[73] Anderson B.R., Gunawidjaja R., Eilers H. (2015). Photodegradation and self-healing in a Rhodamine 6G dye and Y_2_O_3_ nanoparticle-doped polyurethane random laser. Appl. Phys. B.

[74] van Staveren H.J., Moes C.J.M., van Marie J., Prahl S.A., van Gemert M.J.C. (1991). Light scattering in lntralipid-10% in the wavelength range of 400–1100 nm. Appl. Opt..

[75] Spinelli L., Botwicz M., Zolek N., Kacprzak M., Milej D., Sawosz P., Liebert A., Weigel U., Durduran T., Foschum F. (2014). Determination of reference values for optical properties of liquid phantoms based on Intralipid and India ink. Biomed. Opt. Express.

[76] Di Ninni P., Martelli F., Zaccanti G. (2011). Intralipid: Towards a diffusive reference standard for optical tissue phantoms. Phys. Med. Biol..

[77] Aernouts B., Zamora-Rojas E., Beers R.V., Watté R., Wang L., Tsuta M., Lammertyn J., Saeys W. (2013). Supercontinuum laser based optical characterization of Intralipid^®^ phantoms in the 500–2250 nm range. Opt. Express.

[78] Aernouts B., Beers R.V., Watté R., Lammertyn J., Saeys W. (2014). Dependent scattering in Intralipid^®^ phantoms in the 600–1850 nm range. Opt. Express.

